# Multifrequency Investigation of Single- and Double-Stranded DNA with Scalable Metamaterial-Based THz Biosensors

**DOI:** 10.3390/bios12070483

**Published:** 2022-07-01

**Authors:** Christian Weisenstein, Merle Richter, Anna Katharina Wigger, Anja K. Bosserhoff, Peter Haring Bolívar

**Affiliations:** 1 Institute of High Frequency and Quantum Electronics HQE, University of Siegen, 57076 Siegen, Germany; merle.richter@uni-siegen.de (M.R.); anna.wigger@uni-siegen.de (A.K.W.); peter.haring@uni-siegen.de (P.H.B.); 2 Institute of Biochemistry and Molecular Medicine, Friedrich-Alexander-University Erlangen-Nürnberg, 91054 Erlangen, Germany; anja.bosserhoff@fau.de

**Keywords:** metamaterial, DNA, THz, terahertz, biosensor, biomolecular, sensitivity, FSS, biomolecules, sensor

## Abstract

Due to the occurrence of THz-excited vibrational modes in biomacromolecules, the THz frequency range has been identified as particularly suitable for developing and applying new bioanalytical methods. We present a scalable THz metamaterial-based biosensor being utilized for the multifrequency investigation of single- and double-stranded DNA (ssDNA and dsDNA) samples. It is demonstrated that the metamaterial resonance frequency shift by the DNA’s presence depends on frequency. Our experiments with the scalable THz biosensors demonstrate a major change in the degree of the power function for dsDNA by 1.53 ± 0.06 and, in comparison, 0.34 ± 0.11 for ssDNA as a function of metamaterial resonance frequency. Thus, there is a significant advantage for dsDNA detection that can be used for increased sensitivity of biomolecular detection at higher frequencies. This work represents a first step for application-specific biosensors with potential advantages in sensitivity, specificity, and robustness.

## 1. Introduction

As resonance frequencies in the THz range are associated with macro- and biomolecular interactions [1,2], this frequency range is considered suitable for the development of new bioanalytical methods. The first THz experiments with label-free detection of biomolecules had already been demonstrated in the early 2000s [3,4,5,6]. However, the sensitivity of spectroscopic THz investigations as used in these early experiments is limited since the wavelength at THz frequencies (300 μm at 1 THz) is typically orders of magnitude larger than the size of the investigated biomolecules (<10 nm). This leads to the fact that, for many real-world bioanalytic applications, macroscopic state-of-the-art THz setups are either not sensitive enough, lack in performance, or do not show the required selectivity in bioanalyte detection when compared to established biosensing technologies. Consequently, various methods and concepts have been introduced in recent years to improve the interaction of sample and analyzing THz radiation. Metamaterial-based THz biosensors have proven to be particularly suitable, as these biosensors are relatively easy to fabricate using standard lithographic processes and significantly enhance sensitivity for biomolecular detection [7,8]. The ability to immobilize biomolecules on the surface of these biosensors represents a further important step towards their application as competitive biosensors [9,10].

A biosensor, in a general sense, is typically designed for the detection of a specific biomolecule such as a tumor marker. It is therefore advantageous to design and adapt the biosensor in such a way that the sensitivity is maximized for exactly one specific analytic task. However, to date, the focus of THz-biosensor development has been solely on improving the sensitivity of metamaterials and not on optimizing the biosensor with respect to a particular analyte. More precisely, most THz metamaterial-based biosensors generally offer a highly sensitive, but non-specific, solution to an analytic task.

Amino acids and nucleotides are of particular physiological relevance, as they represent the building blocks of more complex molecules, such as proteins and DNA, and are widely investigated with THz analytic methods. A large number of vibrational modes can be excited by THz waves within and between these molecules [11]. In the past, many THz investigations of DNA have been performed in aqueous environments with relatively large sample densities and volumes, in order to gain insights into the characteristics of theses molecules. These include, among others, the dielectric response of molecular solutions [12,13], modification of the molecular structure and spectral signatures as a function of both the polarization state of incident radiation [14] and the hydration level [15].

Only in the recent past have research activities started investigating DNA with metamaterial-based biosensors. These are primarily focused on enhancing the sensitivity of DNA detection, e.g., with a THz metamaterial biosensor based on gold nanoparticles [16] or graphene-combined nanoslot-based terahertz sensors [17]. Other works address the mutation of DNA bases, for example, with microfluidic structures [18] or with terahertz-attenuated total-reflection microfluidic cells [19], while binding experiments are also performed [20]. Various metamaterial-based sensors for DNA detection have been presented; however, to our knowledge, there is no scalability approach and investigation of DNA, as presented in this work.

The approach we introduce in this work addresses the idea of a THz biosensor based on scalable asymmetric double split ring resonators (aDSRR), which is specialized for DNA-sensing applications such as tumor marker detection. We present three different biosensor layouts using aDSRR as sensing elements with identical shapes but scaled in their geometric dimensions to realize resonance frequencies of 0.3 THz, 0.6 THz and 1 THz. A constant sample amount of ssDNA and dsDNA proportional to the sensor area was applied on these biosensors under identical conditions. We demonstrate that the frequency shift as a function of resonance frequency of the aDSRR differs significantly for ss- and dsDNA. This allows to enhance the detection capability of the binding state of DNA for a high resonator frequency in comparison with lower resonator frequencies and thus represents a first step in the development of molecule- or application-specific metamaterial-based biosensors.

## 2. Materials and Methods

### 2.1. Structure

**Figure 1:**
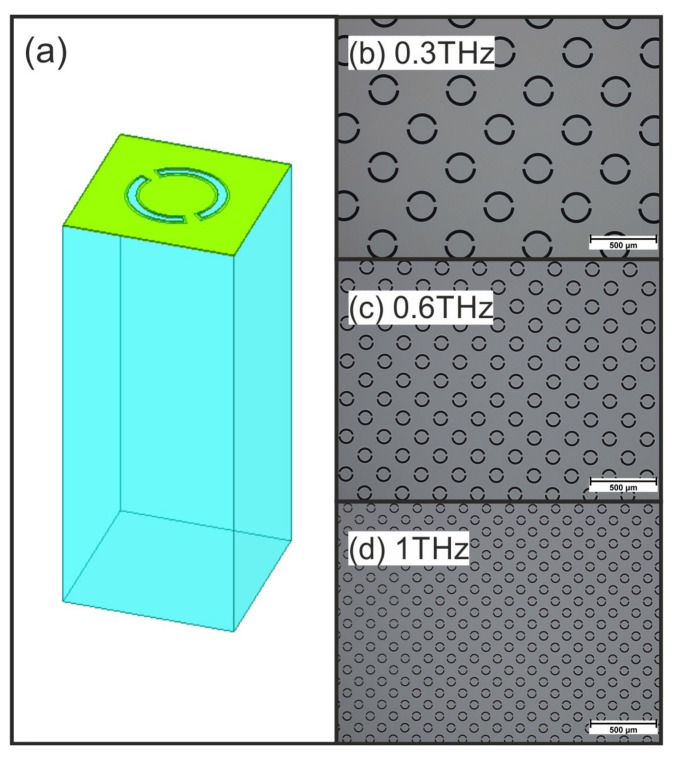
(**a**) Simulation model of the aDSRR structure (for 0.6 THz). The structure is scaled from 0.3 THz to 0.6 THz by a factor of 2.2 and from 0.6 THz to 1 THz by a factor of 1.7. Microscopic images of the fabricated structures for (**b**) 0.3 THz, (**c**) 0.6 THz and (**d**) 1 THz.

The schematic structure of the proposed scalable biosensor is shown in Figure 1a, for the simulation model with a resonance frequency of 0.6 THz. Microscopic images of aDSRR arrays with resonance frequencies of 0.3 THz in Figure 1b, 0.6 THz in Figure 1c and 1 THz in Figure 1d. We have chosen these three frequency ranges as they are sufficiently far apart to provide information about the frequency dependency and also provide favorable spectroscopic analysis windows. Each biosensor consists of the metamaterial structure in form of aDSRR, which was introduced in our previous publication [10]. This metallic structure is deposited on an h${}_{\mathrm{Sub}}$ = 500 μm fused silica substrate. Each of the three biosensor types is designed in such a way that an array of aDSRR forms a field on which a measurement query can be performed. The biosensor for 0.3 THz consists of 16 query fields, each about 3.7× 3.7 mm${}^{2}$ in size. The biosensors for 0.6 THz and 1 THz consist of 25 query fields, each of which is about 2.9×2.9 mm${}^{2}$ in size. The query fields for 0.3 THz are larger than for 0.6 THz and 1 THz because the focal diameter in the measurement setup is significantly larger at this frequency. Each of the query fields is separated by a trench etched into the substrate, so that solutions dropped onto the query fields do not mix.

### 2.2. Fabrication

The aDSRR structures were fabricated by metallization on top of the 500 μm fused silica UV grade substrate. The THz refractive index of fused silica was n ≈ 1.96 and remained constant in the THz range [21]. The metallization was based on a 10 nm chromium layer which acted as adhesive agent for the 200 nm gold layer. An additional 10 nm chromium layer was used as surface passivation. The metal was structured using standard photolithography and wet-etching processes. Subsequently, a wet-etching process with hydrofluoric acid was utilized in order to perform a 3 μm deep isotropic undercut etch into the quartz substrate. This etching process creates a freestanding metal structure, which was used for the field separation trenches. This allows a selective functionalization, which was not used in this work, but has already been presented in detail [10]. Microscopic images show the fabricated aDSRR arrays for 0.3 THz in Figure 1b, for 0.6 THz in Figure 1c and 1 THz in Figure 1d.

### 2.3. Dimensioning

**Figure 2:**
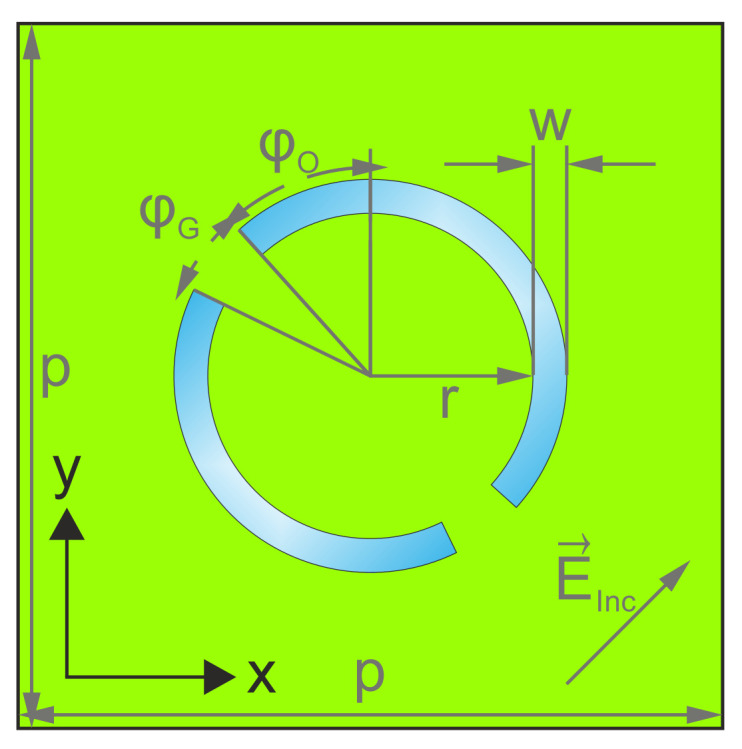
aDSRR unit cell with dimensioning.

The metamaterial aDSRR structure, i.e., two curved slits with different lengths in the metal surface, is depicted in Figure 2. The basic structure has been presented previously [10], but has now been extended to provide a frequency-scalable biosensor platform. We show and explain the design parameter exemplary for the biosensor with a resonance frequency of 0.3 THz, as the dimensional parameters for 0.6 THz and 1 THz are reciprocally linearly scaled with frequency.

The unit cell periodicity for the 0.3 THz structure is *p* = 416 μm, the ring radius is *r* = 96 μm and the width of the two arcs is *w* = 20 μm. The design is symmetrical to a line at a 45${}^{\circ }$ angle between the *x*- and *y*-axes. The lengths of the two arcs are defined by the offset angle $\varphi_{O}=42^{\circ }$ and the gap angle $\varphi_{G}=22^{\circ }$. The wave vector $\vec{E}$ in Figure 2 denotes the polarization of the incident wave normal to the sensor surface. The design parameters are summarized in Table 1. As the cell periodicity decreases with increasing frequency, the array size *A* increases from 61 elements for the 0.3 THz biosensor to 145 elements for 0.6 THz and to 421 elements for 1 THz.

The sensing mechanism of the frequency scalable aDSRR finds its origin in the Fano resonance of the two asymmetric slits in the metal surface. The fundamental principle can be explained on the basis of the positive aDSRR structure, which consists of two asymmetric arcs typically made of metal. An antiparallel current flows in these two metal arcs at the resonant frequency, caused by the interaction of the asymmetric arcs. This principle is commonly known under the term Fano resonance [22], but also explained as trapped modes [23], electromagnetically induced transparency (EIT) [24], resonance hybridization model [25] and antenna pair interaction [7]. By applying Babinet’s principle, the positive structure can be transformed into the complementary structure, assuming perfect conductors and infinitely thin metal layers [26,27]. Thereby, the reflection is interchanged with transmission and electric field with magnetic field, resulting in a 90${}^{\circ }$ change in polarization direction.

### 2.4. Simulation and Material Model

For the simulation and modeling of the scalable aDSRR structures, we used the 3D electromagnetic simulation software Ansys HFSS Electronics Desktop 2021R1. Each aDSRR unit cell was simulated as a repetitive element with periodicity *p*, periodic boundary conditions and Floquet port excitation. The material parameter for the simulation model for gold were calculated from the complex dielectric function of the Drude model, resulting, for the dielectric constant, in $\varepsilon_{r}=-1.12\times 10^{5}$ and, for the conductivity, in σ = 4.01 $\times \;10^{7}\;$S/m [28]. The quartz substrate was modeled with $\varepsilon_{r}$ = 3.81 and a dielectric loss tangent of δ = 0.0001 [21,29]. The material parameters for DNA were gained from comparative experimental approaches and resulted in a layer thickness of 0.1 μm, a dielectric constant of $\varepsilon_{r}=2.6$, a bulk conductivity of σ = 100 kS/m and a dielectric loss tangent of δ = 0.001 [30]. The parameters specified here were obtained from previous studies with a functionalized DNA film and are therefore not directly transferable to the experiments in this work, especially in terms of film thickness. We adjusted the DNA layer thickness, therefore, to 1 μm.

### 2.5. Measurement Setup and Data Processing

In order to detect small frequency shifts as a result of the biosensor measurements with DNA, we utilized the TeraScan 1550 system from TOPTICA Photonics AG. This system provides a THz frequency-domain platform with high bandwidth, high dynamic range and a spectral resolution of 1 MHz [31], which allows the detection of small frequency shifts. The system uses thermal tuning of telecommunication-distributed feedback (DFB) lasers and InGaAs-based photomixers for CW-terahertz generation and detection. We used this system to build up a quasi-optical transmission setup for the biosensor measurements with off-axis parabolic mirrors and motorized stages for precise positioning of the biosensors and each query field in the center of the transmission focus. Each frequency sweep was set in such a way that the resonance frequency of each biosensor was in the center of the sweep range. We set the step size to 40 MHz and the integration time to 10 ms.

The processing of the obtained data is described in the following. The measured photo current in the THz detector is in the form of a cosine and directly proportional to the THz electric field. As this cosine signal only contains a real part, we used the Hilbert transform for the calculation of complex data. After this, we appled an inverse fast Fourier transform (IFFT) to transform the frequency-domain signal into the time domain. The measured signal contains many oscillations which typically have a higher frequency than the sample signal. These oscillations were eliminated by the implementation of a tapered cosine window. This filter sets all signals to zero, except for the main signal, and calculates a cosine-weighted transition from the measured signal to the zero signal outside the window function. FFT is then used to transform the data back to the frequency domain. Finally, we normalized the data to an air measurement. Similar data handling can also be found in further literature [32].

To obtain statistically relevant data, each of the 25 (for 0.6 THz and 1 THz biosensors) or 16 (for the 0.3 THz biosensor) query fields was measured five times in succession. From each of these measurements, the Fano resonance parameters relevant for the analysis were determined. The arithmetic mean and the standard error of the mean (SEM) were then calculated from the five measurements of the parameter values. Since the query fields can differ slightly in resonance frequency due to deviations in fabrication, a complete measurement of the biosensor was carried out after each biochemical process step. From this, different characteristic values were calculated, for example, the frequency shift in a query field was then determined by comparing the position of the resonance feature of two measurements of one query field. The calculation of the error of this difference is based on the maximum error. Details of the error calculations are described in Appendix A Equations (A1)–(A4).

Reference fields were loaded with deionized water (DI water) to provide a monitoring capability during each process step. Reference measurements are essential to control and monitor changes that may occur due to unwanted chemical or mechanical effects. Changes in the reference fields were averaged and subtracted from the measurement fields.

### 2.6. Sample Preparation

#### 2.6.1. Materials

Tris(2-carboxyethyl)phosphine hydrochloride (TCEP), 95%, 0.5 mol/L in H${}_{2}$O was purchased at Alfa Aesar and used without further purification. Water was purified until it reached a conductance of ≤0.058μS/cm before use and is therefore designated as DI water. DNA oligomers were synthesized by Eurofins Genomics, Ebersberg and used without further purification.

tssDNAsequence (AP1/cJun): 5’thi-CGCTTGATGAGTCAGCCGGAA-3’

ssDNA sequence (AP1/cJun): 5’-TTCCGGCTGACTCATCAAGCG-3’

#### 2.6.2. Procedures

dsDNA solution: TCEP (1 μL, 1 mmol/L solution in DI water) was added to a solution of tssDNA (500 pmol) in DI water (11.5 μL). ssDNA (500 pmol) solved in DI water (12.5 μL) was combined with the tssDNA solution and homogenized for 45 min at 25 °C in a thermal shaker. The resulting stock solution ($c_{d1}$ = 20 μmol/L) was diluted with DI water in order to obtain solutions with the concentrations $c_{d2}$ = 10 μmol/L, $c_{d3}$ = 5 μmol/L, and $c_{d4}$ = 1 μmol/L.

ssDNA solution: TCEP (1 μL, 1 mmol/L solution in DI water) was added to a solution of tssDNA (500 pmol) in DI water (24 μL). The resulting stock solution ($c_{s1}$ = 20 μmol/L) was diluted with DI water in order to obtain solutions with the concentrations $c_{s2}$ = 10 μmol/L, $c_{s3}$ = 5 μmol/L, and $c_{s4}$ = 1 μmol/L. The composition of the diluted ssDNA and dsDNA solutions are summarized in Appendix C Table A1.

**Figure A1:**
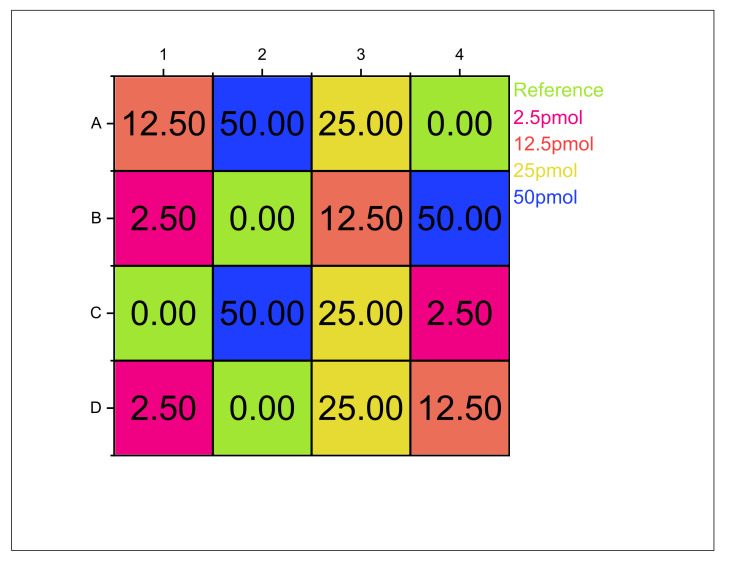
Occupation scheme for the 0.3 THz biosensors. The number in each query field indicates the deposited amount of substance for each field.

**Figure A2:**
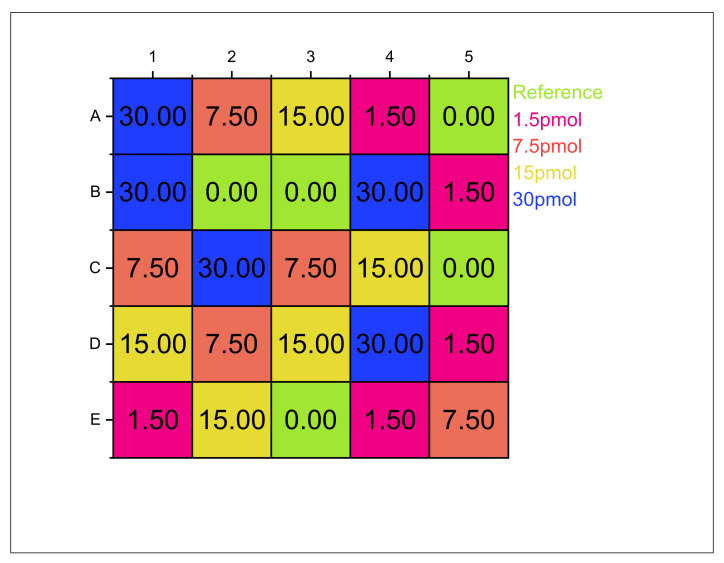
Occupation scheme for the 0.6 THz and 1 THz biosensors. The number in each query field indicates the deposited amount of substance for each field.

For THz measurements, a solvent casting [33] technique was used, in which a small volume of the corresponding DNA solution or DI water as reference sample was placed each on a query field of the sensor (see Appendix B Figure A1 and Figure A2 for occupancy scheme) and the solvent was removed under reduced pressure at 20–22 °C in order to form a solid DNA film. As the query fields for the 0.3 THz biosensors are larger in area than the 0.6 THz and 1 THz biosensors and in order to obtain a similar DNA film thickness, the resulting sample droplet sizes are 2.5 μL and 1.5 μL, respectively. The DNA film thickness was measured with the stylus profiler Bruker DektakXT.

## 3. Results

### 3.1. Simulation and Measurement Comparison

**Figure 3:**
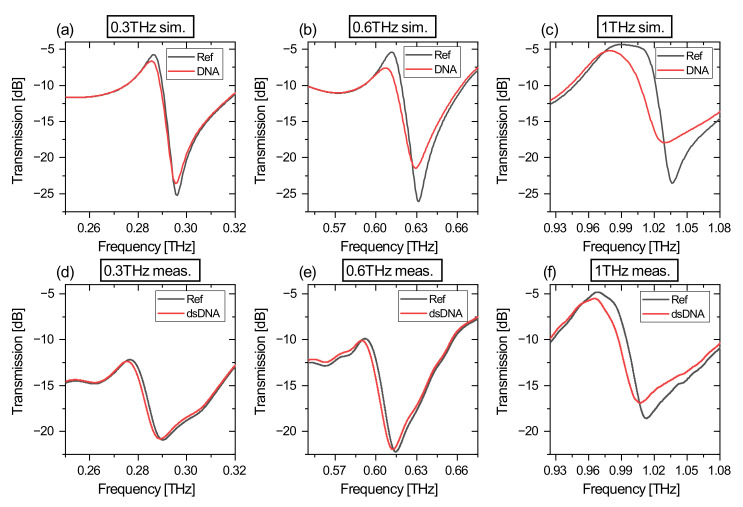
Comparison of simulation (upper row) and measurement results (bottom row). For each diagram, the reference is indicated in black and for DNA in red. (**a**) shows the simulation result for 0.3 THz biosensors, (**b**) for 0.6 THz and (**c**) for 1 THz. The measurement results of the biosensors are shown for (**d**) 0.3 THz, (**e**) 0.6 THz and (**f**) for 1 THz.

The simulation results for the scalable aDSRR structure are shown in Figure 3a–c, respectively, for the three different frequency ranges. Each simulation distinguishes between the empty reference case (Ref) and the case with a DNA layer (DNA). For the three simulation models, a typical Fano-resonance double-resonance feature (DRF) can be observed in the transmission spectra. The peak-to-peak transmission difference (PPTD) and the shape of the resonance for the reference case is at a comparable level for all three models. The 1 THz biosensor shows a broadened transmission maximum of the DRF resulting from the Farbry–Perót resonance of the substrate. When we compare the reference and the DNA-layer simulation results, a shift in the resonance frequency and a reduction in the PPTD is observed, which is a direct result of the dielectric loading of the resonators with DNA. The changes in these properties have a nonlinear relationship as a function of the resonance frequency and will be discussed in detail in the next sections. As a consequence of the attenuated PPTD, the maximum negative slope of the DRF is reduced. Comparing the simulation results with the measurement results with respect to the PPTD attenuation, the assumed DNA-layer thickness seems to be not fitted perfectly, as the PPTD reduction in the measurements is smaller compared to the simulation. A summary and comparison with the measurement results is shown in Table 2. Here, the maximum negative slope at higher resonance frequencies is reduced, while the PPTD remains at the same level, which leads to a broader DRF.

The measurement results for the scalable aDSRR structure are depicted in Figure 3d–f, for the empty reference case (Ref) and for the case with 50/30 pmol dsDNA (dsDNA), respectively. Similar to the simulation results, a typical DRF is observed for the three biosensor layouts. The shape and the relative PPTD are similar for the three biosensor models, except for the 0.3 THz biosensor, which has a smaller PPTD compared to the other biosensors. It is considered that this results from the relative transmission amplitudes, which are changing as a function of frequency and the absolute transmitted power of the measurement system decreases for higher frequencies. Furthermore, signal reflections in the THz path are more significant at lower measurement frequencies than at higher measurement frequencies and can also have an additional negative influence on the DRF amplitude. Comparing each biosensor in the reference case and loaded with 50/30 pmol dsDNA, we observe a nonlinear relationship of the frequency shift as a function of the resonance frequency, as is expected from the simulation model. The maximum negative slope of the DRF changes by loading with DNA, which is compared to the simulation results in Table 2.

### 3.2. Parameter Analysis

**Figure 4:**
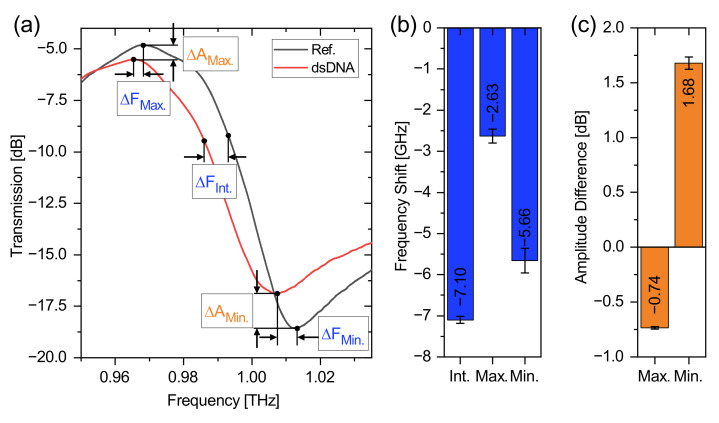
Parametric evaluation of the DRF, using an example measurement with the 1 THz biosensor with 30 pmol dsDNA. The transmission spectra of the reference and dsDNA measurement are featured in (**a**). The extracted parameters are highlighted in (**b**,**c**). (**b**) shows the frequency shift as a result of the dsDNA loading at the intersection point of the slope $\Delta F_{\mathrm{Int}.}$, the maximum position $\Delta F_{\mathrm{Max}.}$ and minimum position $\Delta F_{\mathrm{Min}.}$. (**c**) shows the amplitude difference between the reference and the dsDNA at the maximum position $\Delta A_{\mathrm{Max}.}$ and minimum position $\Delta A_{\mathrm{Min}.}$.

To quantitatively evaluate the effects of loading DNA on the three biosensors, we first investigate an exemplary measurement of the 1 THz biosensor loaded with 30 pmol dsDNA (cf. Figure 4a). This acts as an example analysis, which is representative for all measurements that were examined in the exact same fashion. We first define three characteristic positions in the DRF of the Fano resonance: (i) The maximum position at which the frequency shift $\Delta F_{\mathrm{Max}.}$ and the amplitude change $\Delta A_{\mathrm{Max}.}$ are analyzed. (ii) The minimum position at which the frequency shift $\Delta F_{\mathrm{Min}.}$ and the amplitude change $\Delta A_{\mathrm{Min}.}$ are analyzed. (iii) An intersection point of the slope of the resonance at which the frequency shift $\Delta F_{\mathrm{Int}.}$ can be observed. This intersection point is at 0.5 in the linear transmission spectrum, which has been normalized to 0/1 to minimize influences of amplitude changes.

The changes in frequency shift are summarized in Figure 4b and the changes in amplitude are shown in Figure 4c. From Figure 4b, we obtain a frequency shift of −7.10 ± 0.08 GHz for the intersection, −2.63 ± 0.17 GHz for the maximum- and −5.66 ± 0.30 GHz for the minimum position. The large difference in the magnitude of the frequency shift between minimum and maximum is clearly evident, while the shift between minimum and intersection point is at a comparable level. Furthermore, the standard deviation of the frequency shift at the intersection point is lowest with approx. 80 MHz, while that of the frequency shift at the minimum position is largest with approx. 300 MHz, as the signal-to-noise ratio is at a minimum at this point. Since the frequency shift is largest and the standard deviation is smallest for the intersection point, subsequent investigations will be based exclusively on this parameter. This already represents an interesting finding, since the frequency shift is typically determined at the minimum position of the resonance feature [20,34]. The conclusions drawn from this analysis can be applied to the three biosensor designs and remain valid for all frequency ranges.

Additionally, Figure 4c shows changes in amplitude of the maximum and minimum transmission. Thus, for the maximum position, we observe a decrease in amplitude by −0.74± 0.01 dB, while at the minimum position, the amplitude increases by 1.68± 0.06 dB. What was already true for the frequency shift is also true for the amplitude: The larger relative change is observed at the minimum position. The change in amplitude causes a reduction of the PPTD and is thus responsible for the reduction in the slope when loading the biosensor with DNA. This is attributable to the dielectric losses of DNA, which reduces the Q factor of a resonator [23]. We concentrate, in the following, on the frequency shift at the intersection point which represents the most significant and reliable parameter.

### 3.3. Frequency-Dependent Frequency Shift of ss- and dsDNA

**Figure 5:**
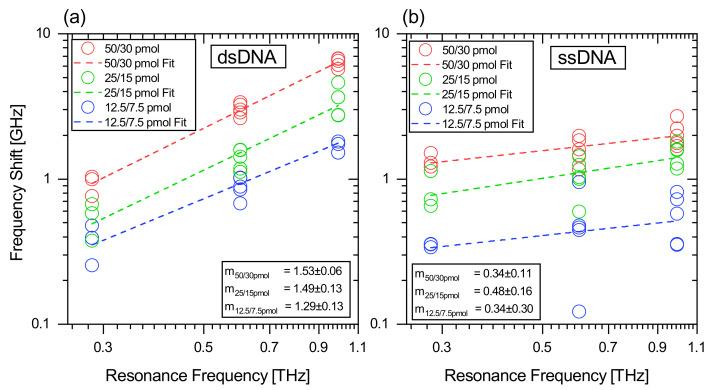
Negative frequency shift as a function of the resonance frequency for (**a**) dsDNA and (**b**) ssDNA. The frequency shift is fitted with a linear function on the double logarithmic scale and indicated in dashed lines for sample amounts of 50/30 pmol in red, for 25/15 pmol in green and for 12.5/7.5 pmol in blue. The parameter *m* describes the slope of the fit function. The first number of the amount of substance indicates the used sample material for the 0.3 THz sensors and the second number for 0.6 THz and 1 THz, respectively.

In the following, we compare the frequency shift at the intersection point for ssDNA and for dsDNA for different sample amounts for the three biosensor variants, as depicted in Figure 5. Due to the logarithmic representation, the shift to lower frequencies caused by the loading of the sensor is shown as a positive number in this figure. To obtain statistically reliable results, each sample configuration was measured on five different query fields on one biosensor (for 0.6 THz and 1 THz) or on three different query fields (0.3 THz), for the substance amounts 50/30 pmol (red), 25/15 pmol (green) and 12.5/7.5 pmol (blue). The sample amount 2.5/1.5 pmol was also measured but not evaluated, as the sample amount was too low and produced results with less significance and clarity. Since each query field was measured five times, each measuring point in Figure 5 is subject to error. However, no error bar is depicted for reasons of clarity.

The results of this experiment for dsDNA are shown in Figure 5a and for ssDNA in Figure 5b in log–log plots. We choose this representation as the differences between the two DNA conformations are highlighted well and, more importantly, it allows to extract the degree of the power function which is a result of the relationship between the frequency shift and resonance frequency of the biosensors. For the experiment with dsDNA, the exemplary presented maximum frequency shift for the 1 THz biosensor is −6.78 ± 0.08 GHz, for the 0.6 THz biosensor −3.38 ± 0.02 GHz and for the 0.3 THz biosensor −1.04± 0.01 GHz with the sample amount of 50/30 pmol. For ssDNA, the exemplary presented maximum frequency shift for the 1 THz biosensor is −2.71 ± 0.05 GHz, for the 0.6 THz biosensor −1.99 ± 0.01 GHz and for the 0.3 THz biosensor −1.52 ± 0.01 GHz with the sample amount of 50/30 pmol. It should be noted that each amount of substance was measured independently on five (three) query fields of each biosensor and deviations in the measured frequency shift can occur due to the manual processing. For the 25/15 pmol and 12.5/7.5 pmol sample amounts, it is observed that the frequency shifts are reduced for the three biosensor variants. This occurs as the DNA-layer thickness is decreasing for smaller sample amounts.

We then analyzed the frequency shifts caused by DNA as a function of the resonance frequency of the biosensors with a fit function. These fit functions are depicted as dotted lines in Figure 5 and appear as linear functions in the log–log plot. We have used the linear function y=a+m·x for the fit, where *a* represents the vertical intercept and *m* is then the slope of the function. Calculating this function into a linear coordinate system results in $y\;=\;A\cdot x^{m}$. Here, *A* describes the coefficient of the power function and is not considered further in the analysis, while the exponent *m* defines the degree of the power function. From this conversion, it becomes clear that, from the slope *m* in the log–log plot, the degree of the power function can be derived. It is found that *m* undergoes a major change when we compare ss- and dsDNA. For the dsDNA we observe a slope of $m_{50/30\mathrm{pmol}}$ = 1.53 ± 0.06, $m_{25/15\mathrm{pmol}}$ = 1.49 ± 0.13 and $m_{12.5/7.5\mathrm{pmol}}$ = 1.29 ± 0.13, while for ssDNA it was $m_{50/30\mathrm{pmol}}$ = 0.34 ± 0.11, $m_{25/15\mathrm{pmol}}$ = 0.48 ± 0.16 and $m_{12.5/7.5\mathrm{pmol}}$ = 0.34 ± 0.30. Transferred to the degree of the power function, this results in a dependence for dsDNA of about $x^{3/2}$ (for the maximum sample amount only) and for ssDNA of about $x^{1/3}$. From these results, three important findings can be derived. (i) The significant difference in the slope allows the direct differentiation between ss- and dsDNA as a function of the resonance frequency. (ii) For the three tested sample amounts, the slope *m* remains close to the same value, which proves the reliability of the measurement methods and procedures. (iii) With a Pearson R correlation coefficient of 0.9917–0.9460 for dsDNA, these results indicate a strong-to-perfect linear relation between frequency shift and resonance frequency at the log–log scale [35]. Only for ssDNA does the Pearson R drop to strong to moderate (0.6955–0.325), as the absolute frequency shifts are smaller, which results in larger deviations.

## 4. Discussion

The comparison between simulation and measurement results reveals differences in the PPTD and in the frequency shift when loading the biosensors with DNA. Nevertheless, the simulation results represent the basic response in a good way. The shape of the resonance and its steepness is influenced by many factors. These include the finite size of the aDSRR arrays used, the limited conductivity of the metals, the variations between design and fabricated structures, signal reflections in the THz path, and the frequency-dependent performance of the measurement system used. The modeling of DNA is also significantly different compared to our previous works, since the DNA layer is not functionalized as a monolayer, but is applied as a solid film instead. Furthermore, the DNA film is simulated without frequency dependence based on the initial assumption, that the DNA material parameters remain constant in the observed frequency range. However, the results of this work show that this assumption is not valid.

In order to understand the origin of the frequency shift and the absorption due to the DNA loading of the biosensors, we focus our discussion on the analysis of the change in the slope and amplitude and, in particular, on the frequency shift. From the measurement analysis summarized in Table 2, it is clear that there is a significant change in the maximum negative slope due to the loading with dsDNA. This change is related to dsDNA only and originates from the reduction in the maximum amplitude and an increase in the minimum amplitude. Experience from analyzing the Q factors for lossy THz metamaterials [23] suggests that the observed changes result from induced losses by the DNA sample. This statement is supported by previous studies with hybridized and denatured DNA, where a frequency-dependent change in transmission was exclusively observed for hybridized DNA [3].

By analyzing the frequency shift, we consider three parameters of the DRF: maximum-, minimum- and intersection-point, chosen to be equidistant to the maximum and minimum points with regard to signal intensity. For the evaluation, we decided to focus on the intersection point, as this frequency shift, in general, delivers greater insights with small error, when compared to the other two characteristic attributes. The investigation of the maximum position does not provide any further information for the comparison of ss- and dsDNA, since the frequency shift for both samples is at comparable levels. The reason for this behavior is not yet fully understood. If we consider the dependence of the frequency shift at the intersection point for dsDNA as a function of the resonance frequency, we can conclude that the degree of the power function with *m* > 1 results from a refractive index of dsDNA that remains constant as a function of frequency in the observed range. This statement is supported by the simulation results, which use a DNA model that is not frequency-dependent and also achieves a degree with *m* > 1. However, since the DNA model used in the simulation was not intended to be applied as a bulk DNA-layer model, the results should be treated as an approximation, which allows a quantitative evaluation only. The *m* < 1 observed for ssDNA allows the assumption that the refractive index is reduced as a function of frequency. However, it should be noted at this point that further investigations are necessary to understand the origin of the differences between the two DNA sample conformations. However, the results already point at interesting differences in the THz properties as a function of hybridization state, indicating the stark advantages of the THz sensing of biomolecular binding, which is very attractive for robust biomolecular recognition analytics.

At this point, it should be noted that the molecular weight of ssDNA amounts to only half of the molecular weight of dsDNA. Moreover, short oligomers such as the ones that are used in this work, tend to denature when dissolved in DI water. It is reasonable to assume a certain hybridization effect upon solvent evaporation, as can be deduced from [36,37]. In order to interpret the resonance frequency shift and amplitude data more precisely, the DNA-layer thicknesses on the sensor surface were traced with a stylus profiler. The approximate volume of each film was derived from these profiles. It was found that the volume of the ssDNA films amounts to 17% of the corresponding dsDNA film. However, the films were found to be shaped inhomogeneously, resulting from the solvent casting method that was used in the formation of the samples. To minimize the influence of the DNA film thickness inhomogeneity, an average film thickness was used for the calculation of the volume and five (three) independent experiments were performed with each sample amount on each biosensor. The significant difference in volume between ssDNA and dsDNA could possibly originate from the different flexibility of single strands as opposed to double strands [38]. This can lead to differences in density considering the short time in which the film formation took place, which would not allow for crystallization to occur. At the same time, ssDNA tends to bind more water molecules per nucleobase than dsDNA [37]. Although the measurements were conducted after evaporating the solvent, it is certain that some moisture remained in close vicinity to the DNA molecules and thus influenced the refractive index of the DNA layer and the shift in resonance frequency. No indication of this effect on the measurement results can be given at this point, but it is clear that low absorption as a function of frequency is evident for ssDNA, which then indicates lower absorption of the ssDNA itself.

A direct comparison with state-of-the-art THz biosensors is a difficult task at this point, since there are only few comparable works with similar sample types. For example, the work of Yang et al. [16] describes an extremely sensitive THz biosensor with a limit of detection (LOD) of 2.77 fM but employs gold nanoparticles and rolling circle amplification on a THz metamaterial surface and is therefore not directly comparable. In the work of Shih et al. [20], an ATP aptamer is detected at a minimum concentration of 1 μM using a nanofluidic THz biosensor. In comparison, the sample resulting from the minimum concentration we measured is 1 μM (corresponding to the amount of substance 2.5/1.5 pmol) at a comparable level. However, we would like to note that the minimization of the LOD was not the aim of this work and can only be considered on the side. A more detailed description about the potential sensitivity of our biosensor and how extremely low amounts of substance can be detected by methods such as selective functionalization has been shown in our previous work [10].

It is generally proven that a larger amount of a dielectric load leads to a larger shift in resonance frequency. This can be seen separately for dsDNA data and for ssDNA data by comparing the different sample amounts. When comparing dsDNA with ssDNA it becomes apparent that, especially at lower resonance frequencies, ssDNA generates a distinct shift in the DRF which is much larger than could be expected. We assume that this might result from a frequency-dependent refractive index of the ssDNA, as the interaction with the THz radiation is typically lower for ssDNA compared to dsDNA. The different volumes of ssDNA and dsDNA films are further parameters that have to be taken into account when analyzing the frequency shifts. However, by varying the sample amount within the same DNA conformation, the thickness of the DNA film was also modified. In the analysis of the fit parameters, however, only an influence on the amplitude of the frequency shift is observed, which is represented by parameter *A* in the fit function. The influence on the degree of the power function, parameter *m* of the fit function, is weak. Nevertheless, it can be concluded that biosensors with high resonance frequencies, such as 1 THz, are suited very well to distinguishing between ssDNA and dsDNA, leading to promising applications in biochemical analysis.

## 5. Conclusions

The aim of this work, which includes the development, fabrication and application evaluation of a scalable THz biosensor system, was successfully achieved. With the detailed DRF parameter analysis, the calculated intersection point method provides benefits in the detection of small frequency shifts with high accuracy, which plays an important role in the application of biosensors for the detection of minute amounts of biomolecular substances. With the presented measurement results of ssDNA and dsDNA with THz biosensors operating at resonance frequencies of 0.3 THz, 0.6 THz and 1 THz, we worked out the difference in the degree of the power function which is a result of the relationship between frequency shift and resonance frequency. A major change in the degree of the power function was observed, which was at a maximum of 1.53 ± 0.06 for dsDNA, compared to a maximum of 0.48 ± 0.16 for ssDNA. This significant difference allows the direct identification of the DNA binding state as a function of the biosensors resonance frequency. To our knowledge, such a multifrequency analysis in the THz range has not been performed before. The frequency shift in dsDNA detected by this method, which depends on the biosensor’s resonance frequency, is an important finding for the application in THz biosensors. However, further investigations of this behavior are necessary for a detailed understanding of the nature of the frequency shift and absorption by ssDNA and dsDNA. Collectively, our work represents a first step towards applying specific THz biosensors, which enables potentially significant advances in sensitivity, specificity and robustness for biomedical applications.

## Figures and Tables

**Table 1:** Geometric parameters of the aDSRR biosensors with resonance frequencies of 0.3 THz, 0.6 THz and 1 THz.

Frequency	0.3 THz	0.6 THz	1 THz
Cell size *p*	416 μm	202 μm	124 μm
Ring radius *r*	96 μm	46 μm	29 μm
Slot width *w*	20 μm	10 μm	6 μm
Offset angle $\varphi_{O}$	42°	42°	42°
Gap angle $\varphi_{G}$	22°	22°	22°
Array size *A*	61	145	421

**Table 2:** Comparison of the maximum negative slope of the DRF for the simulation and measurement results for biosensors with resonance frequencies of 0.3 THz, 0.6 THz and 1 THz.

	Simulation		Measurement	
Freq. / THz	Ref. / dB/GHz	DNA / dB/GHz	Ref. / dB/GHz	dsDNA / dB/GHz
0.3	−3.18	−2.69	−0.90	−0.87
0.6	−1.57	−1.00	−0.86	−0.78
1	−1.15	−0.52	−0.61	−0.48

## Data Availability

The data presented in this study are available on request from the corresponding author.

## Abbreviations

The following abbreviations are used in this manuscript:

DNA	Deoxyribonucleic acid
ssDNA	Single stranded DNA
dsDNA	Double stranded DNA
aDSRR	Asymmetric double split ring resonator
UV	Ultraviolet
EIT	Electromagnetically induced transparency
HFSS	High-frequency structure simulator
DFB	Distributed feedback
CW	Continuous wave
FFT	Fast Fourier transform
IFFT	Inverse FFT
SD	Standard deviation of the mean
SEM	Standard error of the mean
DI	Deionized
TCEP	Tris(2-carboxyethyl)phosphine hydrochloride
DRF	Double resonance feature
PPTD	Peak-to-peak transmission difference
LOD	Limit of detection

## Appendix A. Error Calculation

Each of the 25 (0.6 THz and 1 THz biosensors) and 16 (0.3 THz biosensor) query fields was measured five times in succession, in order to obtain statistically relevant data. The parameter resonance frequency, and minimum and maximum amplitude were determined from each measurement. From this, the arithmetic mean was determined for each parameter:

(A1) $$\begin{array}{cc} \bar{x} & =\frac{1}{N}\sum_{i=1}^{N}x_{i}\\ \; & =\frac{x_{1}+x_{2}+\cdots +x_{N}}{N}. \end{array}$$

*N* denotes, here, the number of measurements, and $x_{i}$ is the resonance parameter. With the help of the mean, we could then calculate the standard deviation of the mean (SD):

(A2) $$\begin{array}{cc} \sigma_{x} & =\sqrt{\frac{1}{N-1}\cdot \sum_{i=1}^{N}{\left(x_{i}-\bar{x}\right)}^{2}}\\ \; & =\sqrt{\frac{1}{N-1}\cdot \left[{\left(x_{1}-\bar{x}\right)}^{2}+{\left(x_{2}-\bar{x}\right)}^{2}+\cdots +{\left(x_{N}-\bar{x}\right)}^{2}\right]}, \end{array}$$

which defines the standard deviation of a single measured value of the mean. As we are interested in the deviation of the real value, we then calculated the standard error of the mean (SEM):

(A3) $$\sigma_{\bar{x}}=\frac{1}{\sqrt{N}}\cdot \sigma_{x}.$$

All quantities subject to error within this work are reported as SEM values. If the difference in two values with errors was calculated, we always assume the maximum error, which is defined as follows:

(A4) $$\begin{array}{cc} \Delta z & =\sum_{j=1}^{m}|\frac{\delta f}{\delta x_{j}}\cdot \Delta x_{j}|\\ \; & =|\frac{\delta f}{\delta x_{1}}\cdot \Delta x_{1}\left|+\right|\frac{\delta f}{\delta x_{2}}\cdot \Delta x_{2}\left|+\cdots +\right|\frac{\delta f}{\delta x_{m}}\cdot \Delta x_{m}|. \end{array}$$

## Appendix B. Biosensor Occupation Scheme

Two basic biosensor designs were made, one for the 0.3 THz biosensors and one for the 0.6 THz and 1 THz biosensors. The main difference is the number of query fields, which are 16 and 25, the size of each query field, which are about 13.69 mm${}^{2}$ and 8.41 mm${}^{2}$ and the number of aDSRR elements, 61 (0.3 THz), 145 (0.6 THz) and 421 (1 THz). The query fields are arranged in a checkerboard pattern, where the row is numbered with consecutive letters and the column with a consecutive number. Figure A1 shows the design layout for the 0.3 THz biosensors and Figure A2 for the 0.6 THz and 1 THz biosensors. The number in each query field indicates the amount of substance used and is distributed randomly in order to avoid systematic errors. The sample amounts are 50 pmol, 25 pmol, 12.5 pmol and 2.5 pmol for the 0.3 THz biosensor, while the 0 pmol fields were loaded with DI water as a reference. The amount of substance was adjusted for the 0.6 THz and 1 THz biosensors as the area of the query fields was reduced and to achieve a comparable DNA film thickness. This results in an amount of substance of 30 pmol, 15 pmol, 7.5 pmol and 1.5 pmol for the 0.6 THz and 1 THz biosensor, while the 0 pmol fields were loaded with DI water as a reference. As was mentioned in the main article, the 2.5/1.5 pmol experiments were not used for further analysis, as this sample amount attained the limit of detection.

## Appendix C. DNA Sample Composition

The ssDNA and dsDNA diluted solutions were prepared as indicated in Table A1. The subscript *d* represents the dsDNA samples and the subscript *s* represents the ssDNA samples, each at four different concentrations, *c*. In addition to the amounts of substance *n* for tssDNA and ssDNA, TCEP was added to the solution used to deprotect the DNA. The total volume of the solution is denoted by *V*.

**Table A1:** Composition of DNA samples including sample concentration *c*, amount of substance *n* and volume *V*.

Sample	c/μmol L${}^{-1}$	$n_{\mathrm{tssDNA}}\;/\;$pmol	$n_{\mathrm{TCEP}}\;/\;$pmol	$n_{\mathrm{ssDNA}}\;/\;$pmol	V/μL
$c_{d1}$	20	500	1000	500	25
$c_{d2}$	10	250	500	250	25
$c_{d3}$	5	125	250	125	25
$c_{d4}$	1	25	50	25	25
$c_{s1}$	20	500	1000	0	25
$c_{s2}$	10	250	500	0	25
$c_{s3}$	5	125	250	0	25
$c_{s4}$	1	25	50	0	25
